# Comprehensive Analysis of the Structure and Function of Peptide:N-Glycanase 1 and Relationship with Congenital Disorder of Deglycosylation

**DOI:** 10.3390/nu14091690

**Published:** 2022-04-19

**Authors:** Xiangguang Miao, Jin Wu, Hongping Chen, Guanting Lu

**Affiliations:** 1 Queen Mary School, Nanchang University, No. 1299 Xuefu Avenue, Honggutan New District, Nanchang 330036, China; wujin170827@gmail.com; 2 Laboratory of Translational Medicine Research, Department of Pathology, Deyang People’s Hospital, No. 173 First Section of Taishanbei Road, Jingyang District, Deyang 618000, China; jin.wu@roswellpark.org; 3 Deyang Key Laboratory of Tumor Molecular Research, No. 173 First Section of Taishanbei Road, Jingyang District, Deyang 618000, China; 4 Department of Molecular & Cellular Biology, Roswell Park Comprehensive Cancer Center, Elm and Carlton Streets, Buffalo, NY 14263, USA; 5 Department of Histology and Embryology, Medical College of Nanchang University, Nanchang 330006, China

**Keywords:** N-glycosylation, NGLY1, ER associated degradation process, congenital disorder of deglycosylation, NFE2L1

## Abstract

The cytosolic PNGase (peptide:N-glycanase), also known as peptide-N4-(N-acetyl-β-glucosaminyl)-asparagine amidase, is a well-conserved deglycosylation enzyme (EC 3.5.1.52) which catalyzes the non-lysosomal hydrolysis of an N(4)-(acetyl-β-d-glucosaminyl) asparagine residue (Asn, N) into a N-acetyl-β-d-glucosaminyl-amine and a peptide containing an aspartate residue (Asp, D). This enzyme (NGLY1) plays an essential role in the clearance of misfolded or unassembled glycoproteins through a process named ER-associated degradation (ERAD). Accumulating evidence also points out that NGLY1 deficiency can cause an autosomal recessive (AR) human genetic disorder associated with abnormal development and congenital disorder of deglycosylation. In addition, the loss of NGLY1 can affect multiple cellular pathways, including but not limited to NFE2L1 pathway, Creb1/Atf1-AQP pathway, BMP pathway, AMPK pathway, and SLC12A2 ion transporter, which might be the underlying reasons for a constellation of clinical phenotypes of NGLY1 deficiency. The current comprehensive review uncovers the NGLY1’ssdetailed structure and its important roles for participation in ERAD, involvement in CDDG and potential treatment for NGLY1 deficiency.

## 1. Introduction

In living eukaryotes, secretory proteins are translocated co-translationally into the endoplasmic reticulum (ER) lumen via signal recognition particles (SRPs) and subjected to a quality control process. During this process, proteins are modified and sorted in ER based on their three-dimensional conformation. The correctly folded proteins are then transferred to the Golgi apparatus for maturation and rerouted to other cellular compartments via transport vesicles. The misfolded or unassembled proteins are subjected to degradation through a process known as ER-associated degradation (ERAD) [1].

The cytosolic PNGase (peptide:N-glycanase; Png1 in yeast; pngl in fruit fly; NGLY1/Ngly1 in human/mice), also known as peptide-N4-(N-acetyl-β-glucosaminyl)-asparagine amidase, is a well-conserved deglycosylation enzyme (EC 3.5.1.52) which catalyzes the non-lysosomal hydrolysis of an N(4)-(acetyl-β-d-glucosaminyl) asparagine residue (Asn, N) into N-acetyl-β-d-glucosaminyl-amine and a peptide containing an aspartate residue (Asp, D) [2]. NGLY1 plays essential roles in clearance of misfolded glycoproteins via releasing intact N-glycans from N-glycosylated proteins. Accumulating evidences point out that NGLY1 deficiency can cause an autosomal recessive (AR) human genetic disorder with abnormal development, indicating its critical functions for the normal development of mammals [3,4]. The current review focuses mainly on NGLY1’s structure, participation in ERAD, involvement in CCDD, and potential treatment for NGLY1 deficiency. 

## 2. The Discovery of Peptide:N-Glycanase

**Figure 1:**
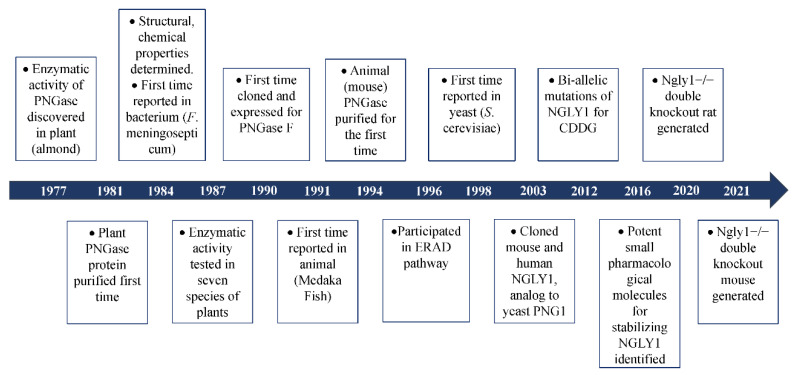
Brief history of the discovery of PNGase.

The glyco-peptidase, partially purified from the seed emulsion of a fruit plant (almond, *Prunus dulcis*), was first reported in 1977 in order to show its enzymatic activity to cleave β-aspartyl-glycosyl-amine linkages in glyco-peptides [5]. Later, this glyco-peptidase was discovered in several other plants, including jack bean (*Canavalia ensiformis*) [6], split pea (*Pisum sativum*) [7], thale cress (*Arabidopsis thaliana*) [8], lentil (*Lens culinaris*), pinto bean (*Phaseolus vulgaris*), lima bean (*Phaseolus limensis*), barley (*Hordeum vulgare*) and wheat (*Triticum vulgare*) [7]. This implied that the existence of this specific glyco-peptidase might be ubiquitous in the plant kingdom. In 1978, some basic enzymatic characters of this new glyco-peptidase such as Km value, optimum pH, and inhibitors were revealed [9]. Since the enzyme could only hydrolyze N-glyco-peptides longer than three amino acids [9], it was named peptide:N-glycosidase (PNGase). It was reported in 1981 that PNGase (PNGase A for almond) could highly efficiently cleave short ovalbumin- and bromelain-derived N-glyco-peptides which were modified with a high content of mannose and complex oligosaccharides [10]. Interestingly, it was discovered in the same year that the carbohydrate removal of pepsin-digested glycoproteins by PNGase was almost three times higher than that of the intact untreated proteins [11]. This implied that the complex oligosaccharides might hinder the accessibility of PNGase to the cleavage site of the target glycoproteins in their native state. Treating glycoproteins with heat in sodium dodecyl sulfate (SDS) or high concentrations of chao-tropic salts such as NaSCN and NaClO_4_ with disulfide bond reducing agent β-mercapto-ethanol (BME) to denature the native structures, more N-glycans were released from the denatured and unfolded proteins upon treatment with PNGase [12]. In 1984, the enzymatic activity of PNGase was discovered in the Gram-negative bacterial pathogen for meningitis and septicemia, *Flavobacterium meningosepticum*, and was named PNGase F [13]. This was the discovery of PNGase in prokaryotes. More importantly, in 1989, free di-N-acetyl-chitobiose based oligosaccharides were detected in the extract of eggs of trout (*Plecoglossus altivelis*) [14], dace (*Tribolodon hakonensis*) and flounder (*Paralichthys olivaceus*) [15]. Since di-N-acetyl-chitobiose was mainly produced from N-linked glycoproteins by PNGase, it is quite reasonable to infer the presence of PNGase in the animal kingdom. This led to the discovery of the Peptide:N-Glycosidase in the embryos of Madaka fish (*Oryzias latipes*) in 1991 [16]. Two years later, PNGase was discovered in mouse (L-929, BALB-3T3 and P3X63-Ag8.U1) and human (TIG-3S) cell lines [17]. In 1997 and 1998, PNGase was identified in two fungi, *Aspergillus tubigensis* [18] and *Saccharomyces cerevisiae* [19], respectively. Up to now, the existence of PNGase has been found in the plant, prokaryotes, fungi, and animal kingdoms (Figure 1). The biochemical characteristics of some PNGases are listed in Supplementary Table S1. This PNGase was named Png1 in *Arabidopsis thaliana*, Png1 in yeast, png-1 *Caenorhabditis elegans*, Pngl in fruit fly, Ngly1 in mice and rat, and NGLY1 in humans. For readability, the gene is termed NGLY1/Ngly1 in the whole article.

Based on experimental and bio-informatic analysis, Ngly1 is universally expressed in almost all living cells. Loss-of-functional (LoF) mutations of Ngly1 might be detrimental to the deglycosylation process. In 2012, bi-allelic compound mutations of NGLY1 were identified by whole-exome sequencing (WES) in a boy with congenital anomalies and/or intellectual disabilities [3]. This was the first time that NGLY1 was associated with a specific disorder. Since the disorder was caused by pathologic mutations in the specific enzyme responsible for deglycosylation, the disease was named congenital disorder of deglycosylation (CDDG) or NGLY1-congenital disorder of deglycosylation (NGLY1-CDDG; OMIM#615273). With the discovery of mutations in NGLY1 for CDDG [3,4,20,21,22], more and more researches are concentrated on the molecular mechanisms of NGLY1 in the process of deglycosylation (Figure 1). 

## 3. Protein Structure of Non-Animal Ngly1s

**Figure 2:**
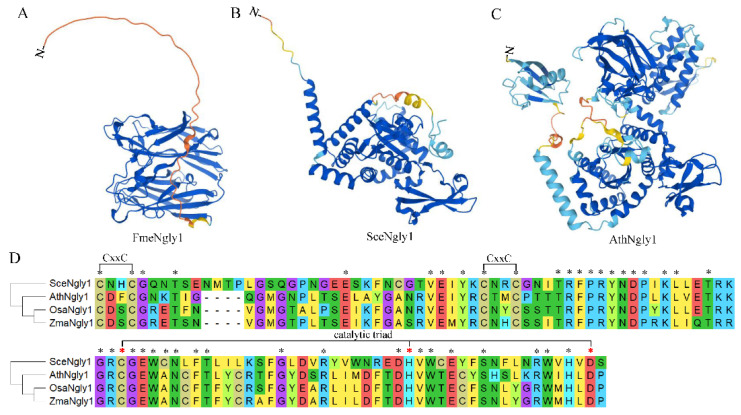
Predicted structures for non-animal Ngly1 and the core transglutaminase-like domain. (**A**) AlphaFold predicted structure of FmeNgly1; (**B**) AlphaFold predicted structure of SceNgly1; (**C**) AlphaFold predicted structure of AthNgly1; (**D**) Sequence alignment of the core transglutaminase-like domain. Fme, *Flavobacterium meningosepticum*; Sce, *Saccharomyces cerevisiae*; Ath, *Arabidopsis thaliana*; Osa, *Oryza sativa* Japonica; Zma, *Zea mays*; * represents conserved residuals; red * represents the three residuals of the catalytic triad.

It has been reported that Ngly1 exists in almost all species, from single-cell bacteria, fungi, to multi-cell sophisticated mammals. As for PGNase F from *Flavobacterium meningosepticum*, it was the first cloned bacterial peptide:N-glycosidase and named FmeNgly1 temporarily [23]. The FmeNgly1 protein consisted of 354 amino acids with a molecular weight of 39.03 kD. It contained a secretory signal motif at the N-terminal (1–40 amino acids) [24] and was secreted into the culture medium during growth [25]. The remaining sequence (41–354) was folded into an active enzymatic domain which consisted of two eight-stranded antiparallel β-sandwich subdomains (subdomain A from 41–177, subdomain B from 183–354) (Figure 2A). The two subdomains were packed side by side with parallel principal axes to form a binding cleft for substrates [26]. It has been reported that three acidic residues, Asp100, Glu158 and Glu246, were located at the interface between subdomain A and B, essential for enzymatic activity. The crystal structure of FmeNgly1 revealed that the N,N’-diacetyl-chitobiose molecule of the substrate was inserted edgewise into the cleft and spanned the distance between two acidic residues (Asp100 and Glu158). Glu246 did not contact directly with the N-glycan substrate, but contributed importantly to the stabilization of the reaction intermediates [27]. It has been verified that the Asp100Asn mutant had no detectable enzymatic activity, while Glu206Gln and Glu118Gln maintained less than 0.01% and 0.10% of the wild-type enzymatic activity, respectively [27]. This FmeNgly1 enzyme could release a broad range of N-linked glycans including high mannose, complex (bi-, tri-, and tetra-), hybrid, and poly-sialic, regardless of the periphery of the oligosaccharide [23]. 

As a single-cell model eukaryote, yeast (*Saccharomyces cerevisiae*) has been used extensively to study the molecular function of numerous proteins. The yeast-derived PGNase (PNG1, SceNgly1) contains 363 amino acids with a molecular weight of 42.49 kD. Unlike FmeNgly1, SceNgly1 is soluble without a secretory signal peptide or transmembrane motif, and mainly localizes in the nucleus, and at a lower level in the cytosol [28]. The protein has a Zn-binding domain, the core transglutaminase (TGase) domain containing a Cys-His-Asp triad, and the Rad23-binding domain (Figure 2B,D) [29,30]. For SceNgly1, the Zn-binding domain contained four Cys residues (Cys-129, -132, -165, and -168) forming two CxxC motifs (Figure 2D), which possessed the ability to bind zinc metals. Disruption of the CxxC motifs by EDTA to chelate Zn^2+^ or Dithiothreitol (DTT) in order to reduce the bisulfide bonds could destroy the deglycosylation activity of SceNgly1 [30]. The core TGase enzymatic domain was composed of six β-strands, which were maintained by three α-helices and several loops. A deep cleft was produced between the two subdomains formed by the strands and helix combinations. According to the binding specificity, this cleft could be divided into a carbohydrate-binding region and a protein-binding region. As for the Rad23-binding domain, it was formed by the two N-terminal and two C-terminal α-helices, which was essential for the deglycosylation of the denatured or mis-folded form of the N-linked glycoproteins through 26S proteasome-mediated degradation [30,31,32]. It has been reported that, although lacking a specific domain for binding mannose, SceNgly1 still had a substrate priority toward high mannose-type carbohydrate chains [33]. Removal of the C-terminal disordered tail (329–363) could result in significant decrease of the enzymatic activity (about 10-fold), implying that this region might be responsible for the favorability of the mannose residues [29]. Variations in PNGase activity, free oligosaccharide (fOS) generation and PNG1 expression have been described as a function of yeast growth: SecNgly1’s enzyme activity [19], generation levels of fOS [34] and expression of SecNgly1 [35] were increased at the end of the exponential phase of growth and reached maximal level during the stationary phase of growth [35].

As for the plant PGNase, the protein AthNgly1, derived from the model plant *Arabidopsis thaliana*, was taken to elaborate the protein structures. In 2001, AthNgly1 was identified by bioinformatic analysis as a single-copy gene encoding for a putative PGNase [36]. AthNgly1 contains 721 amino acid residues with a molecular weight of 82.45 kD. According to the Conserved Domain Database (CDD) [37], AthNgly1 is composed of three domains, a N-terminal β-grasp ubiquitin-like fold (16–63 aa), a core transglutaminase domain with catalytic Cys-His-Asp triad (199–296 aa) and a C-terminal small acidic domain of XPC-like domain (280–347 aa) (Figure 2C,D). Unlike Ngly1 in other species, AthNgly1 possesses a typical transglutaminase (TGase) activity in a Ca^2+^-, pH-, and GTP-dependent manner [38]. Later, AthNgly1 was verified to contain the PGNase activity also [8] and could substitute, to some extent, for the yeast SceNgly1 to take part in the ERAD via its deglycosylation activity [39]. This indicated that AthNgly1 might contact with Rad23 directly or indirectly to play certain roles in plant ERAD process. 

## 4. General Structures of Animal NGLY1

**Figure 3:**
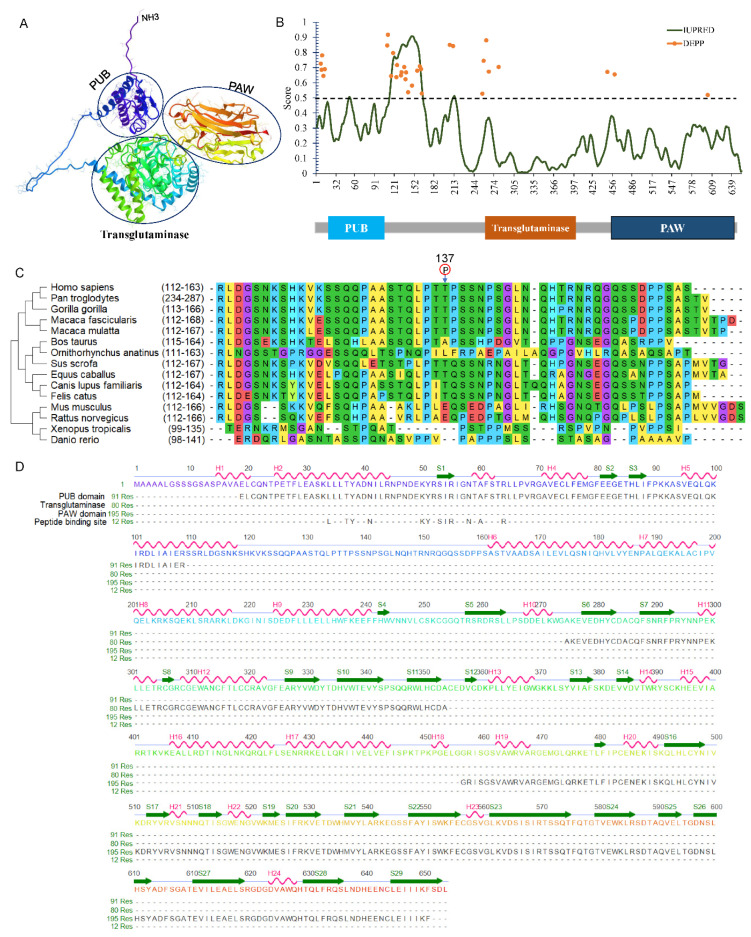
Structural analysis of human NGLY1. (**A**) Three-dimensional structure predicted by AlphaFold; (**B**) Intrinsically-disordered region of NGLY1; (**C**) Alignment of the intrinsically-disordered region; (**D**) Secondary structure of NGLY1.

In the animal kingdom, especially in mammals, the Ngly1 protein commonly contains three separate domains. Take human NGLY1 as an example, the NGLY1 gene (NM_018297) contains 12 exons spanning about 74.39 kb on chromosome 3p24.2. The encoded protein is composed of 654 amino acids with three functional domains, namely, a N-terminal PUB domain, a central transglutaminase-like domain and a C-terminal PAW domain as predicted by AlphaFold (Figure 3A) [40]. A short intrinsically disordered region was identified ranging from 112 to 163 between the PUB and transglutaminase-like domain by IUPred2A [41]. Disorder Enhanced Phosphorylation Predictor (DEEP) revealed a phosphorylation hotspot in this intrinsically-disordered region (Figure 3B). Interestingly, the phosphorylation of Thr137 was identified through a large-scale phospho-proteome analysis [42]. Missense mutations Thr137Pro (c.409A > C) and Thr137Lys (c.410A > G) were detected in a colon adenocarcinoma and liver cancer samples [43]. After comparing the disordered sequences from different species, the Thr residue was strongly conserved in large mammals (Figure 3C), indicating its importance for the function of NGLY1.

N-terminal PUB domain: The PUB (PNGase/UBA or UBX-containing protein) domain, located at the N-terminal (aa 19–109) of NGLY1, was first identified through bioinformatics analysis [36,44]. According to the AlphaFold Protein Structure Database, this PUB domain consists of a bundle of five α-helices that pack onto short three stranded anti-parallel β-sheets (Figure 3D) [45]. Based on evolutionary conservation analysis, PUB domain existed in almost all animals, except for snakes and nematodes. Considering the absence in certain species, it is reasonable to infer that this domain may not be directly involved in the catalytic activity of NGLY1, but rather played other important roles. The ubiquitin-associated (UBA) domain was reported in many ubiquitin-regulatory proteins (UBX), being involved in the ubiquitination pathway, or containing regions homologous to ubiquitin itself, such as CBL, CBLB, MARK3, RAD23A, RAD23B, and UBA2 and UBXN1 [46]. Since the most commonly conserved residues in the UBA domain were non-polar amino acids to stabilize the secondary α-helical structure, it was indicated that the UBA domain was unlikely to be directly involved in phosphorylation or ubiquitination, but rather in mediating protein-protein interactions [36,47,48,49]. The PUB motif could bind to the adaptor protein p97 (also called VCP, Cdc48) which was located on the outer endoplasmic reticulum (ER) membrane [47,50], providing NGLY1 with a molecular platform for the de-glycosylating process toward the retro-translocated misfolded or unassembled proteins [51]. It could also bind with other proteins functioning in the ER-associated degradation (ERAD) pathway, such as DERL1 [52]. 

Central transglutaminase-like core domain: Since it contains the transglutaminase-like core domain, NGLY1 was categorized as a member of the transglutaminase superfamily along with the Cys, His, and Asp catalytic triad, essential for the enzymatic function [2]. The core domain could also be subdivided into a transglutaminase-like region, zinc-binding motif, and RAD23/HR23 binding motif [30,53]. The transglutaminase sequence was responsible for breaking the β-aspartyl-glycosyl-amine linkage between the innermost N-acetyl-glucosamine (GlcNAc) and the Asn residue (N-X-S/T) of the target N-glycoprotein. After surveying the NGLY1 protein sequence, there are four CXXC motifs around or in the transglutaminase-like region. Since the CXXC sequence could bind with Zn^2+^ to form zine-finger motifs, Zn^2+^ might be important for enzymatic activity. It has been reported that several divalent metal ions, such as Zn^2+^, Mg^2+^, Co^2+^, and Cu^2+^ could greatly increase the N-glycanase velocity [54]. However, in most filamentous fungal species such as Neurospora crassa, Cys and His of the catalytic triad in Ngly1 were mutated leading to complete loss of enzymatic activity [55]. This indicated that the N-glycanase activity was not essential for some organisms. The RAD23/HR23 binding motif rendered NGLY1 the ability to bind with Rad23 (RAD23A and RAD23B in humans), thus taking part in the ubiquitin-proteasome pathway [56,57]. Rad23 could also tightly bind with the xeroderma pigmentosum group C (XPC) and participated in the nucleotide excision repair (NER) pathway [58,59]. Therefore, the central transglutaminase core domain of NGLY1 could not only hydrolyze the β-aspartyl-glycosylamine linkage, but also act as adaptor to bind with components in the proteasome or NER pathway. 

C-terminal PAW domain: Compared with the protein structures in different species, a PAW domain was located at the C-terminus of NGLY1. It was named after ‘domain present in PNGases and other worm proteins’. The PAW domain has a β-sandwich architecture composed of two layers containing antiparallel β-strands, and short helices (Figure 3D). The PAW domain was reported to bind with the high content mannose moieties of N-linked oligosaccharide chains [60]. The interaction between PAW domain and the mannose could increase the affinity of NGLY1 for N-glycans to promote enzymatic activity. Combination of transglutaminase core domain and PAW domain contributes to the oligosaccharide-binding specificity of NGLY1 for the N-linked glycans with high mannoses.

## 5. Evolutionary Relationships of NGLY1

**Figure 4:**
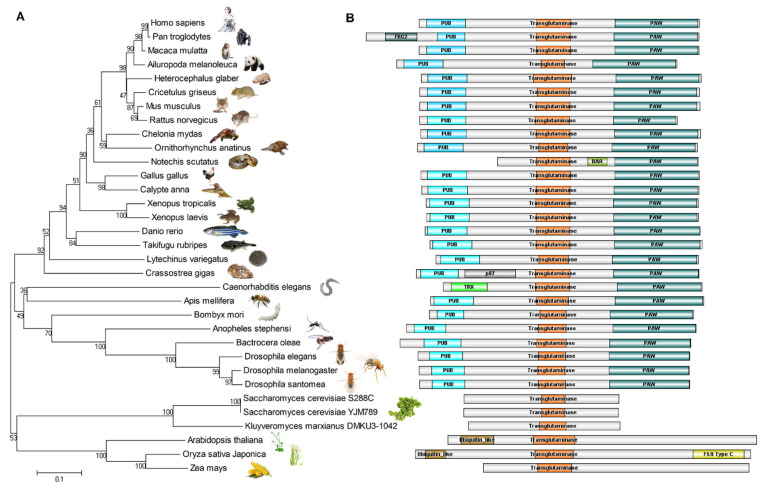
Evolutionary relationships of NGLY1 in 33 taxa. (**A**) Evolutionary phylogeny; (**B**) Protein structures of Ngly1.

The protein sequences of NGLY1 from different species were recruited from the NCBI’s GenBank and UniProt, and aligned by CLUSTALW. The evolutionary phylogeny was constructed using the Minimum Evolution method (Figure 4A) [61]. The structures of these NGLY1s from different species were strongly conserved during evolution (Figure 4B). This implied the importance of the N-glycosylation and de-N-glycosylation system for life, from simple single cells to the most sophisticated mammals. It is clearly shown in the animal kingdom that, except for tiger snake (*Notechis scutatus*) and nematode (*Caenorhabditis elegans*), other NGLY1s contain three domains, a PUB domain, a transglutaminase (TGase) domain and a PAW domain. The Ngly1 protein of tiger snake lacks the PUB domain, but has a BAR (Bin/Amphiphysin/Rvs) motif in the middle of the TGase and PAW domains. It has been reported that the BAR domain possesses the ability to bind with cellular membranes [62,63,64,65], which might substitute for the function of the PUB domain to provide a platform for the de-glycosylating process. The *C. elegans* Ngly1 (png-1) contains a TRX (thioredoxin) domain, instead of the PUB motif. The TRX domain confers Ngly1 with an extra thioredoxin ability [66] and makes Ngly1 a unique bi-functional protein possessing two enzyme activities [67]. It is worth noting that, instead of lacking the specific PUB domain, the Ngly1 in chimpanzee (*Pan troglodytes*) and Pacific oyster (*Crassostrea gigas*) was predicted to have one more FRG2 (cl21160) and Nucleo_P87 (pfam07267) domain, respectively. However, the functions of these two domains are unknown. Experiments should be performed to reveal if these types of Ngly1 are tri-functional proteins. 

In the fungi and plant kingdoms, Ngly1 only contained the transglutaminase (TGase) domain, without the PUB and PAW domains [39]. However, the length of plant Ngly1 was almost two times that of fungi. This implied that the plant Ngly1 might possess more complex functions than fungi. In the dicot plant, *Arabidopsis thaliana*, the Ngly1 has a β-grasp ubiquitin-like fold (cl28922) at the N-terminal. In the monocot plant rice (*Oryza sativa* Japonica), Ngly1 has a β-grasp ubiquitin-like fold (cl28922) at the N-terminal and a F5/8 type C domain (pfam00754) at the C-terminal. The functions of these domains still remain unknown. 

## 6. Roles of NGLY1 in the ERAD Pathway

It has been reported that only the N-linked glycans on short peptides, pepsin-digested, denatured or misfolded glycoproteins could be highly efficiently removed by Ngly1 [11,12]. This implied that the complex N-linked oligosaccharides provide a steric hindrance inhibiting the accessibility of Ngly1 to the hydrolyzation site of the intact target proteins. In most eukaryotic cells, N-linked glycosylation was carried out through a similar cyclic pathway, the dolichol cycle. This started with the generation of a lipid-linked oligosaccharide (LLO) by multiple asparagine-linked N-glycosylation processing enzymes (ALGs) in a stepwise manner [68]. The produced precursor high-mannose oligosaccharide (Glc3Man9GlcNAc2) was then en bloc transferred co-translationally onto the asparagine (Asn, N) acceptor site (N-X-S/T) in nascent polypeptide chains from a dolichyl-linked oligosaccharide donor by the oligosaccharyl-transferase (OST, a multi-protein complex composed of nine subunits in yeast and four subunits in higher eukaryotes) [69,70]. Upon the en bloc transfer, initial trimming of the precursor molecule in ER was carried out by glucosidase I and II (GCSI, GCSII) to remove the last two non-reducing terminal glucose residues from the Glc3Man9GlcNAc2 oligosaccharide [71]. Proteins with the mono-glucosylated glycans (Glc1Man9GlcNAc2) were entered into the calnexin (CNX)/calreticulin (CRT) cycle. CNX or CRT bound with Glc1Man9GlNAc2 to accelerate proper protein folding and prevent its abnormal aggregation [72]. The properly folded proteins were removed from the final glucose residue by GCSII, released from CNX/CRT cycle, and transferred to the cis-Golgi for further processing. Therefore, this trimming step was thought to function as a quality control mechanism in the ER to monitor protein folding. 

However, even under normal physiological conditions, some rapidly synthesized or mutated proteins could be improperly folded (misfolded). The accumulation and aggregation of misfolded proteins would induce cellular toxicity if left unchecked [73,74]. It is well known that the toxic amyloid-like aggregates of β-amyloid (APP), tau (MAPT), α-synuclein (SNCA or PARK1), and islet amyloid polypeptide (IAPP) are linked with neurodegeneration in Alzheimer’s disease (AD), frontotemporal dementia (FTD), Parkinson’s disease (PD) or Type 2 diabetes (T2D) [75]. Therefore, timely clearance of the misfolded or misassembled proteins is essential for physiological cellular homeostasis and cell growth. Fortunately, a quality control system in the ER evolved to pick misfolded oligopeptides out of the properly folded proteins based on their trimmed glycan structures [76].

**Figure 5:**
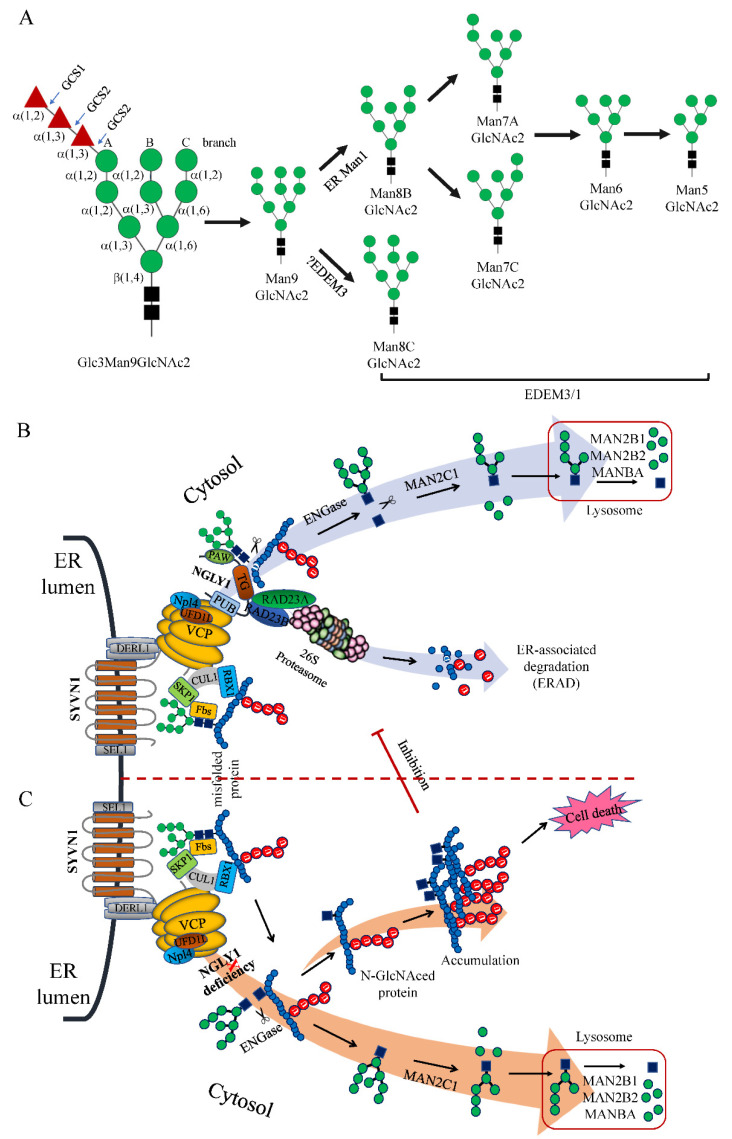
Glc3Man9GlcNAc2 trimming process and ER-associated degradation pathway (ERAD) involving NGLY1. (**A**) Glc3Man9GlcNAc2 trimming process; (**B**) ERAD under normal physiological state; (**C**) ERAD with NGLY1 deficiency.

If the glycoproteins remain misfolded, the terminal mannose residues of the Man9GlcNAc2 glycans could be trimmed by ER resident α-mannosidase 1 (ER Man1) and ER degradation enhancing alpha-mannosidase like proteins (EDEMs) to produce different glycans, most likely M8, M7, M6, and M5 (Figure 5A), which generate a specific signal that flags the glycoprotein for degradation [77,78,79]. The mannose-trimmed misfolded proteins could be recognized and transferred to the Derlin-1 retro-translocation complex (DERL1, SEL1L1 and SYVN1) by several ER chaperones such as HSPA5 (BiP/GRP78), EDEM1, OS9, and ERLEC1 (XTP3-B) [77,80,81,82,83,84]. The picked-out misfolded proteins were retro-translocated into the cytosol by the Derlin-1 retro-translocation complex with the assistance of the energy-providing multi-protein ATPase complex VCP-UFD1L1-NPLOC4 (p97-Ufd1-Npl4) [85,86]. In cytosol, a VCP-binding multi-protein complex Cullin-RING Ligase 1 (CRL1) was formed by a scaffolding cullin protein CUL1, RBX1/Roc1, SKP1 and substrate-binding adaptor F-box proteins (such as FBXO2/Fbs1 and FBXO6/Fbs2), therefore, the complex also being known as SCF (SKP1-cullin-F-box) [87,88]. Upon retrograding into cytosol, the misfolded target proteins with exposed Man5-9GlcNAc2 glycans were bound by FBXO2 or FBXO6 and polyubiquitinated by the ubiquitin ligase of the SCF complex [88,89,90,91]. Subsequently, NGLY1 was hold tightly onto VCP/p97 of the p97-Ufd1-Npl4 complex via its N-terminal PUB domain [47]. Its PAW domain gripped onto the mannose moieties of the polyubiquitinated misfolded proteins and the TGase-like domain cleaved the β-aspartyl-glycosylamine linkage between the innermost N-acetyl-glucosamine (GlcNAc) and the Asn residue (N-X-S/T), releasing a free Man5-9GlcNAc2 and a de-glycated protein with an Asp (D) instead of Asn (N) residue. The processed proteins with Ds were transferred to the 26S proteasome which was linked with NGLY1 via the HR23 complex (RAD23A and RAD23B) for thorough degradation. This distinctive process was named the ER-associated degradation pathway (ERAD) (Figure 5B) [1,92]. The free GlcNAc2 oligosaccharides were hydrolyzed by ENGase (endo-β-N-acetyl-glucosaminidase) to get rid of one GlcNAc [73] and further processed by the cytosolic α-mannosidase (MANC1) to remove a few mannose residues [93]. The processed oligosaccharide was then transferred to the lysosome for thorough degradation by lysosomal α-mannosidase (MAN2B1 and MAN2B2) and β-mannosidase (MANBA) [93,94,95]. 

However, when NGLY1 was deleted or mutated with bi-allelic loss-of-functional mutations leading to NGLY deficiency, the process of ER-associated degradation (ERAD) was interrupted after ubiquitination by SCF ligase complex. The Man5-9GlcNAc2 glycans in the misfolded proteins could not be released by NGLY1; the glycosidic bond between the two GlcNAc residues was cut by ENGase which released a free Man5-9GlcNAc and a protein with a N-GlcNAc residue. With accumulation of the N-GlcNAced misfolding proteins, these might be intertwined together to form insoluble aggregates in the cytosol. The aggregates could inhibit the protein degradation function of the 26S proteasome complex, thus rendering a cellular toxic effect leading to apoptosis of the cells (Figure 5C).

## 7. Alternative Splicing Patterns of NGLY1 in Human and Other Species

**Figure 6:**
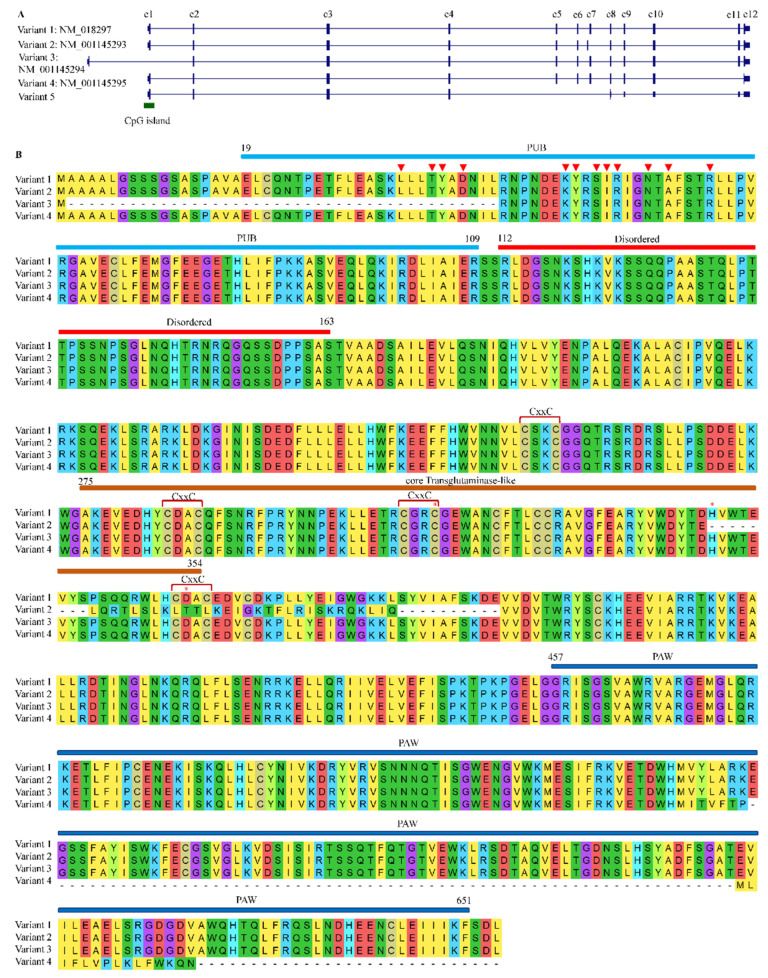
Variants of NGLY1 generated by alternative splicing in humans. (**A**) Transcripts; (**B**) Protein alignments. Red triangles represent peptide binding sites; Red stars the Cys-His-Asp catalytic triad.

After searching reference genomes, no paralogs of NGLY1 were found in animals including humans, therefore, NGLY1 should be a single-copy gene, just as in yeast and plants [8]. Currently, it is widely taken for granted that alternative splicing of eukaryotic transcripts is a common mechanism to generate vast protein diversity from a limited number of genes [96]. Therefore, one may ask whether there exist other NGLY1 isoforms produced through alternative splicing to perform different functions. For humans, five kinds of alternative splicing were identified (Figure 6A). Taking variant 1 (NM_018297) as reference, variant 2 (NM_001145293) used a new exon 7′ instead of the canonical one. The correspondent protein (NP_001138765) lacked the C-terminal part of the TGase core domain without two residues of the Cys-His-Asp catalytic triad (Figure 6B). The variant 3 (NM_001145294) started its transcription at a bi-directional promoter (chr3:25,831,337–25,831,648, shared with OXSM) 6.57 kb upstream of variant 1. The encoded protein lacked part of the PUB domain (19–43) (Figure 6B). Since this portion of PUB contained 4 of the 12 amino acids responsible for binding with VCP (p97), the ER-membrane-bound ability might be significantly impaired. As for the variant 4 (NM_001145295), the exon 11 was skipped to result in a short transcript with 142 bp less. This generated a premature stop codon which in turn shortened the peptide sequence at its C-terminus PAW domain without affecting the PUB and core Transglutaminase-like domain. Since PAW domain was required for the binding of NGLY1 to the high-mannose N-linked oligosaccharides [60], the resultant protein should possess the whole enzymatic activity. However, it was reported that the protein from variant 4 did not seem to be involved in the generation of free oligosaccharides, and therefore in the ERAD process [97]. After browsing the transcripts from NGLY1, a novel variant (variant 5) was identified without three exons (from 5 to 7) which introduced a premature stop codon. This variant might be the target of non-sense mediated mRNA decay (NMD) [98]. More in vitro and in vivo experiments should be carried out to study the functions of these alternatively-spliced transcripts for EARD or non-ERAD pathways. 

**Figure 7:**
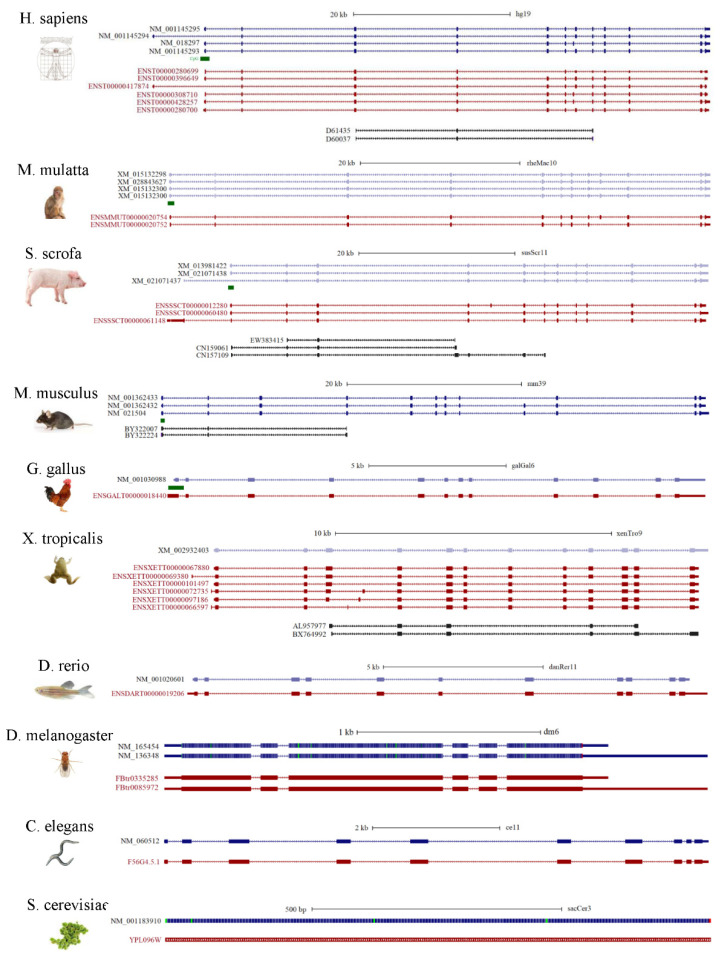
Alternative splicing patterns of NGLY1 in different species.

In order to have a comprehensive understanding of the alternative splicing portrayed for NGLY1 in different species, several model organisms were chosen, since annotations of genomes, genes and their expressions have been relatively well studied. Sequences alignment was performed for the refseqs (reference genes), transcript in Ensembl and mRNAs (or expressed sequence tags) in GenBank from human (*Homo sapiens*), Rhesus monkey (*Macaca mulatta*), Pig (*Sus scrofa*), mouse (*Mus musculus*), Chicken (*Gallus gallus*), tropical clawed frog (*Xenopus tropicalis*), Zebrafish (Danio rerio), fruit fly (*Drosophila melanogaster*), nematode (*Caenorhabditis elegans*), and fungus (*Saccharomyces cerevisiae*) (Figure 7). Interestingly, it was found that, the higher the organism in the phylogeny tree, the more splicing patterns were identified. In yeast, SecNgly1 is a single exon gene and no alternative splicing mRNAs have been reported before now. In nematode, fruit fly and zebrafish, no alternative splicing events involving exons were identified, but only with some alternative polyadenylations. For zebrafish, tropical clawed frog and chicken, Ngly1s span about 15 kb in the genomic regions and only have multiple alternative splicing events involving coding regions. In mouse, two incomplete mRNAs were found to lack the third exon, encoding part of the PUB domain (83–161 aa) and one refseq without the ninth exon. It is worth noting that, in pig, Rhesus monkey and humans, one type of alternative splicing lacking the penultimate exon was identified, which might affect the completeness of the PAW domain. However, its significance and function have not been yet established.

## 8. Different Functional Pathways Participated in by NGLY1

**Figure 8:**
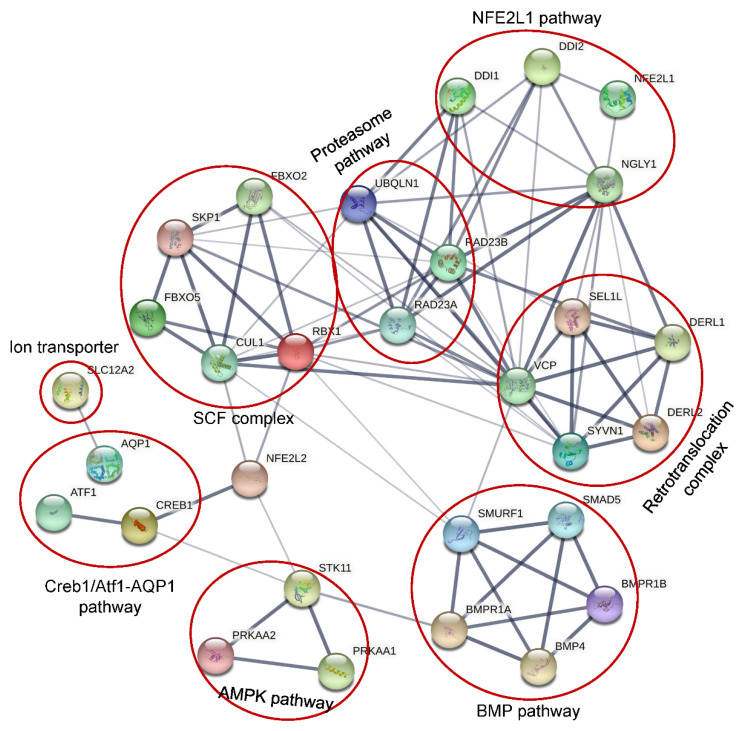
Protein network involving NLGY1 by STRING.

It was widely accepted that NGLY1 played an important role in the ER-associated degradation pathway to remove misfolded glycosylated proteins which were retro-translocated from ER lumen to the cytosol [1,4,74]. Under conditions of NGLY1 deficiency, the misfolded glycoproteins would be accumulated to form insoluble aggregates [4] which could inhibit the activity of 26S and lead to ER stress and cell death [99]. It seemed quite likely that NGLY1 might be involved in a general mechanism to deglycosylate misfolded glycoproteins through the ERAD pathway. However, it has been reported that some proteins could be degraded regardless of glycosylation status [100,101]. In cells with NGLY1 deficiency from Drosophila melanogaster [102] and rat [103], mouse [104] and humans [105], no evidences of ER stress were detected, which was often observed in cells with impaired ERAD function. Several in vivo studies showed that the degradation rate of the majority of ERAD substrates studied was not profoundly altered by a large number of conditions that induced Ngly1 inhibition, such as deletion of the corresponding gene in yeast [28], or use of specific RNAi (RNA interferences) [101], or the inhibitor Z-VAD-FMK (a caspase inhibitor) [106]. This indicated that NGLY1 might play diversified roles in living cells, in addition to being involved in ERAD (Figure 8).

### 8.1. NFE2L1 Pathway

NFE2, like bZIP transcription factor 1 (NFE2L1, also called NRF1) is a transcription factor belonging to the CNC-bZIP family [107]. NFE2L1 is involved in regulation of many cellular functions, such as oxidative stress response, differentiation, inflammatory response, and metabolism. NFE2L1 was ubiquitously expressed and could be induced by cellular stresses such as oxidative stress, ER stress, and inflammation. Under a normal physiological state, the N-glycosylated non-active NFE2L1 was retro-translocated into cytosol for ERAD-dependent degradation by the proteasome [108]. Under cellular stimuli for inhibition of the proteasome function, the retro-translocated NFE2L1 escaped the ERAD and the N-glycans were removed by non-ER bound NGLY1 to make the amino acid transition from Asn (N) to Asp (D) [109]. The deglycosylated NFE2L1 was further cleaved at the Leu104 residue by DNA damage inducible 1 homologs (DDI 1 or DDI 2) to release the ER-bound NFE2L1 from the ER membrane [109,110,111]. The processed NFE2L1 (p110) then entered into the nucleus to activate expression of a subset of proteasome subunits to promote the proteasome function in order to alleviate cellular stresses [107]. However, in NGLY1 deficient cells, the N-linked glycans could not be released, which inhibited the cleavage and activation of NFE2L1. It has been identified that NFE2L1 is highly expressed in the brain, heart, kidney, skeletal muscle, and fat [112]. Disruption of the NFE2L1 pathway caused by NGLY1 mutations might be related to the neurological, renal, skeletal and ophthalmological phenotypes of patients with NGLY1 deficiency.

### 8.2. Creb1/Atf1-AQP Pathway

It has been reported that, regardless of the N-glycanase enzymatic activity, NGLY1 deficiency could decrease transcriptionally the levels of multiple aquaporins (AQPs) by 50–60% in human and mouse cells, indirectly through transcription factors Atf1/Creb1 [104]. Since aquaporins function as a water transmembrane transporter, their decrease could partly explain poor or absent tear production, dry mouth, reduced saliva production [113] and constipation [114]. 

### 8.3. BMP Pathway

In Drosophila, loss of Ngly1 could result in developmental midgut defects, which were similar to the deficiency of BMP signaling [115]. Later, Ngly1 was found to colocalize with endoplasmic reticulum via VCP (p97) to promote the retro-translocation and de-glycosylate the misfolded BMP4 for proteasome degradation, which could increase the efficient traffic of properly-folded BMP4 to its target compartment through the secretary pathway [116]. It has been reported that BMP4 is essential for mesoderm development, limb formation, tooth development, bone induction, nephric duct formation, renal system segmentation and aortic valve morphogenesis [117,118,119]. Mutations of BMP4 could result in eye and brain developmental anomalies [117,120,121], which overlap with NGLY1 deficiency.

### 8.4. AMPK Pathway

In some NGLY1 deficiency patients, altered muscle and liver mitochondrial amount, function and impaired physiology were identified, which could be rescued by restoration of NGLY1 expression, which confirmed the direct relationship of NGLY1 with mitochondrial function [122]. Recently, it has been reported that NGLY1 deficiency could severely reduce the expression and phosphorylation of AMP-activated protein kinase α (AMPKα) in Drosophila larval intestine, mouse embryonic fibroblasts and patient-derived fibroblasts, leading to energy metabolism defects, impaired gut peristalsis, failure to empty the gut, and animal lethality [123]. The reduced AMPKα expression observed in NGLY1 deficiency cells was not caused by the loss of NFE2L1 activity. Restoration of Ngly1 or AMPKα expression could significantly alleviate the energy metabolism defects. 

### 8.5. SLC12A2

Based on lethality association and co-evolution analysis, a conserved Na/K/Cl ion transporter Ncc69 (human NKCC1/2, officially SLC12A1/2) was identified, ubiquitously expressed and associated genetically with Ngly1 in Drosophila [123]. In Ncc69 or Ngly1 knockdown (KD) Drosophila, more than 30% showed severe seizures [124]. In mouse embryonic fibroblasts (MEFs), the homolog of Ncc69, NKCC1 was highly expressed and N-glycosylated, which was important for the function [125]. However, NGLY1 deficiency could disturb the N-glycosylation of NKCC1, which is detrimental to the functionality of the protein. In mammals, NKCC1 is expressed specifically in secretory epithelia, such as salivary, sweat, and lacrimal glands, to promote the basolateral ion absorption and subsequent secretion [126]. The decrease of NKCC1 functionality might account for the alacrima and reduced production of saliva and sweat observed in patients with NGLY1 deficiency.

The variety of functions carried out by this enzyme may explain the diversity and varying severity of symptoms caused by mutations in this gene.

## 9. Mutations for NGLY1-Related Congenital Disorders

In 2012, the first patient harboring pathogenic mutations in NGLY1 were identified by whole exome sequencing (WES) [3]. The bi-allelic mutations of NGLY1 could lead to the rare autosomal recessive congenital disorder of deglycosylation (CDD, OMIM#615273) which was characterized with global developmental delay/intellectual disability (100%), muscular hypotonia (84%), peripheral neuropathy (83%), movement disorder (81%), microcephaly (72%) and poor or absent tear production (76%) [4,20]. Other common features include EEG abnormalities (76%), liver storage or vacuolization (75%), chronic constipation (69%), abnormal MRI (60%), seizures (58%), intrauterine growth delay (55%), strabismus (37%), liver fibrosis (35%), hearing impairment (34%), scoliosis (32%), corneal ulceration or scarring (28%), small hands or feet (28%), ocular apraxia (18%), muscle wasting 10%, and contractures (6%) [20,127,128]. It has been revealed that this disorder mainly affected young children [3,4].

**Figure 9:**
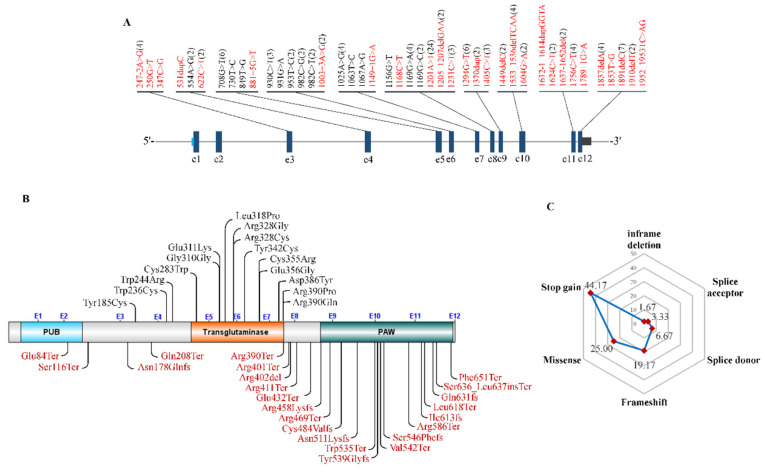
Characterization of mutations of NGLY1. (**A**) mRNA locations; (**B**) Amino acid locations; (**C**) Different types of mutations.

Until now, only 60 patients carrying bi-allelic NGLY1 mutations have been reported in the literature (*n* = 54) and the DECIPHER database (*n* = 6) (Supplementary Table S2). However, according to a personal communication with members of the Grace Science Foundation, more than 100 cases of NGLY1 deficiency have been registered [129], but the personal clinical and genetic data were not shown. 43 different mutations were identified in these patients (Figure 9A), with c.1201A > T (p.Arg401Ter) as the commonest (20%, 24/120) (Figure 9A,B). It is worth noting that nearly all missense mutations were located in the central transglutaminase-like core domain, and stop gain or frameshift in the C-terminal PAW domain (Figure 9C). In these patients, stop gains accounted for 44.17% (53/120), missense 25.00% (30/120), frameshift 19.17% (23/120), splice donor 6.67% (8/120), splice acceptor 3.33% (4/120) and in-frame deletion 1.67% (2/120) (Figure 9C). Nearly all NGLY1 mutations studied thus far are characterized by reduced NGLY1 protein levels and enzymatic activity [130]. 

For the missense mutations, missense3D was used to predict the structural changes introduced by an amino acid substitution based on the AlphFold predicted NGLY1 protein structure (Supplementary Table S3) [45,131]. Except for c.554A > G (p.Tyr185Cys) and c.1067A > G (p.Glu356Gly), other missense mutations possessed the potential to cause structural damage to NGLY1, such as triggering clash alteration (*n* = 1, p.Cys283Trp), altering secondary structure (*n* = 1, p.Arg390Pro), introducing buried Proline (*n* = 2, p.Leu318Pro and p.Arg390Pro), breaking the buried salt bridge (*n* = 2, p.Arg328Cys and p.Arg328Gly), introducing buried charge switch (*n* = 2, p.Glu311Lys and p.Leu318Pro), replacing buried charge residues (*n* = 3, p.Arg328Cys, p.Arg328Gly and p.Arg390Gln), switching buried or exposed state (*n* = 3, p.Cys283Trp, p.Cys355Arg and p.Arg390Pro), breaking buried H-bonds (*n* = 4, p.Arg328Cys, p.Arg328Gly, p.Tyr342Cys and p.Arg390Pro), and expanding cavity volume (*n* = 7, p.Trp236Cys, p.Trp244Arg, p.Leu318Pro, p.Arg328Cys, p.Tyr342Cys, p.Asp386Tyr and p.Arg390Pro) (Supplementary Table S2). For p.Tyr185Cys (c.554A > G), this was identified homozygously in a 3-year-10-month old female (264084) recruited to the DECIPHER database. The phenotypes of this sample include moderate intellectual disability, delayed speech and language development, and echolalia, which was often observed in patients with NGLY1 deficiency [3,20]. It predicted by MutationTaster that this variant could generate an intraexonic splice donor site (GTCT|gtga, score = 0.76) resulting in an in-frame loss of 105 base pairs in exon 4. For c.1067A > G (p.Glu356Gly), it was classed as PM2 (strong) + PP3 (supporting) + PP5 (supporting) and annotated as “Likely pathogenic” according to ACMG classification. The patient also carried a loss-of-function mutation in NGLY1 (c.1201A > T, p.Arg401Ter). Although predicted neutral to the protein structure of NGLY1, no NGLY1 proteins were detected in the muscle and skin fibroblasts of the patient by Western blot [22]. This indicated that the variant p.Glu356Gly could result in protein instability or destruction and be regarded as a disease-causing mutation. Interestingly, nearly absent expressions of NGLY1 mRNAs were detected in fibroblast lines obtained from four patients with bi-allelic NGLY1 mutations [patient 1 (c.1205_1207delGAA, p.Arg402del and c.1624C > T, p.Arg542Ter); patient 2 (homozygous for c.1201A > T, p.Arg401Ter); patient 3 (c.931G > A, p.Glu311Lys and c.730T > C, p.Trp244Arg); patient 4 (c.622C > T, p.Gln208Ter and c.930C > T, p.Gly310=)] [121]. Although two missense mutations in patient 3 were predicted to affect the proper function of NGLY1, they actually impaired the stability of messenger RNAs. In patient 4, c.930C > T was a synonymous mutation (p.Gly310=), but introduced a novel splice donor site. In the lympho-blastoid cell lines from a patient with compound NGLY missense mutations (c.953T > C, p.Leu318Pro and c.1169G > C, p.Arg390Pro), no proteins of NGLY1 were detected (data not shown). Therefore, it seemed quite likely that NGLY1 deficiency resulted not only from nonsense-mediated mRNA decay (NMD) by nonsense or frameshift mutations [98], but also from mRNA or protein instability by missense mutations. This will be verified soon by in vitro and in vivo experiments. 

It might be highly valuable to find out the discrepancy between clinical phenotypes of patients with mutations in different functional domains. However, it is difficult to solve this riddle due to several reasons. First, NGLY1-CDDG is an autosomal recessive disease. Many patients carried different pathogenic mutations in different domains. Second, NGLY1-CDDG is a multisystem disorder, and the clinical phenotypes were not completely recorded, which made the evaluation of disease severity difficult. Third, there might be some unknown genetic modifiers affecting the disease penetrance which could be seen among different patients with the same mutation and same genetic background [4,132]. Finally, in addition to function in ERAD, NGLY1 could functioned in many pathways, such as the NFE2L1 pathway, Creb1/Atf1-AQP pathway, BMP pathway and AMPK pathway (Figure 7). Its combination made this disorder quite difficult to study, and made it difficult to find druggable targets.

## 10. Potential Treatments for NGLY1-CDDG

With the rapid increase of research in NGLY1’s structure, function and cellular pathways for cells and animal models in different species, several molecular methods were tested for the potential to treat NGLY1-deficient diseases.

### 10.1. Exogenous Restoration of NGLY1 Expression

Currently, double-knockout of NGLY1 has been used to study the enzymatic ability and molecular functions of the gene in several model organisms, such as yeast, Drosophila, worms, mouse and rat. After comparing the spectrum of clinical phenotypes and histological analysis, Ngly1^−/−^ rats displayed very similar features to human NGLY1-deficiency patients [103]. Although Ngly1 was ubiquitously expressed, and highly transcribed in brain, lung, heart, kidney, liver, placenta and testis [133], the neurological symptoms in most patients implied the CNS as the most seriously affected organ [128].

AAV9s carrying a copy of human NGLY1 gene (AAV9-hNGLY1) were injected to Ngly1-KO SD rats during the weaning period via intra-cerebro-ventricular (i.c.v.) administration in order to reverse the deterioration of neuronal symptoms [134]. The re-expression of NGLY1 were identified in pons, thalamus, hippocampus, cerebral cortex and cerebellar Purkinje cells, but not detected in liver. The enzymatic activity of NGYL1 in Ngly1^−/−^ rats was restored to a comparable level with that of wildtype controls. AAV9-hNGLY1 was safe and did not lead to liver toxicity. The motor dysfunction caused by NGLY1 deficiency, such as gait abnormalities, motor coordination and balance, were also significantly restored. However, the stride length and grip strength of limbs were not significantly ameliorated, which might be related to the absence of expression of functional NGLY1 in muscles. This was the first time that the expression of NGLY1 in a NGLY1-deficient mammalian model was restored. However, there are many questions to be answered: (1) Whether rat was suitable for studying the underlying molecular mechanisms of NGLY1-CDDG to explore potential drug targets. (2) Since NGLY1 was ubiquitously expressed, whether CNS was the most suitable organ to restore the exogenous NGLY1, and if the injection of AVV9-NGLY1 in more than one organ could ameliorate the clinical phenotypes to a higher degree. (3) Since primates were most closely related to humans in evolution, whether primates with NGLY1 deficiency might be the most appropriate animal model to study the pathogenesis of NGLY1-CDDG and to develop curable drugs.

### 10.2. Targeting ENGase with Small Inhibitors

It has been reported that endo-β-N-acetyl-glucosaminidase (ENGASE) could cleave the glycan moieties from N-linked glycoproteins at the beta-N-acetyl-glucosaminide, releasing a free Man5-9GlcNAc and proteins with a single GlcNAc residue linked to the Asn residual (N-GlcNAc proteins) (Figure 5C) [74]. The N-GlcNAc proteins are prone to aggregate, resulting in the dysfunction of the ERAD process. Additional deletion of ENGASE could partially prevent lethality and alleviate the phenotypes of the Ngly1-loss mice [135]. Therefore, inhibition of ENGASE might be a potential therapeutic site for NGLY1-deficient diseases. Currently, several novel ENGASE inhibitors have been discovered, including Proton Pump Inhibitors (PPIs), Lansoprazole, Rabeprazole and Omeprazole [136]. PPIs could immediately be considered as therapeutic drugs for treating NGLY1-deficiency because of their existing safety and pharmacokinetics profile.

### 10.3. Inhibition FOXB6 (Fbs2)

FOXB6 (also known as Fbs2) is a component of the SCF (SKP1-cullin-F-box) complex and functions as a substrate-binding adaptor (Figure 5B,C) [88]. In NGLY1-deficient cells, FOXB6 was overexpressed and resulted in cytotoxic impairment of the proteasome activity. The overexpressed FOXB6 not only induced ubiquitination of NFE2L1, but also inhibited NFE2L1’s processing by DDI2 and nuclear localizing in cells without NGLY1. Interestingly, additional knockout of Foxb6 could make Ngly1-deficient mice (Foxb6^−/−^; Ngly1^−/−^) viable and exhibit normal motor functions [137]. The survival ratio at P0 was two times higher than in Engase; Ngly1 dKO mice [135]. This indicates that FOXB6 might be a promising drug target for treatment of NGLY1-CDDG. 

### 10.4. Activation of NFE2L2

It has been commonly accepted that inactivation of NFE2F1 was an important factor for the pathogenesis of NGLY1-CDDG. Functional loss of NFE2F1 could result in inhibition of proteasome function, mitochondrial dysfunction and immune dysregulation in NGLY1-deficient cells under proteotoxic stress [138]. In mammals, as a close homologue of NFE2F1, NFE2L2 (NRF2) was responsible for regulation of the expression of similar proteasome subunit genes as NFE2L1 under oxidative stress [139], and the expression of genes for autophagy and mitophagy [140,141]. Unlike the ER-bound NFE2FL1, NFE2L2 was cytosolic and non-N-glycosylated without relying on NGLY1 for its activation [142]. Increased expression of NFE2F2 could promote mitophagy and rescue the mitochondrial and immune homeostasis in Ngly1^−/−^ cells. It has been verified that KEAP1 could strongly bind with and mediate the ubiquitination and degradation of NFE2L2 [143]. Therefore, chemical inhibitors to disrupt the KEAP1-NFE2L2 interaction might be a promising drug target. Interestingly, a natural inhibitor for KEAP1 derived from cruciferous vegetables (such as broccoli, cauliflower, kale, kohlrabi, Brussels sprouts and cabbage), sulforafane could efficiently bind with KEAP1 and robustly increase NFE2L2 protein level to promote the expression of proteasome subunit genes and mitophagy-related genes in NGLY1-deficient cells [138]. The disrupted mitochondria and dysregulated immune response to mtDNAs were significantly decreased after administration of sulforaphane, which indicated that KEAP1-NFE2L2 axis was a novel therapeutic site for correcting the abnormalities of NGLY1 diseases. 

## 11. Perspectives

Research associated with NGLY1 physiology, structure, function and diseases at molecular, cellular and model animal levels has been conducted for almost 50 years, since 1977. However, its alternative splicing patterns, cellular expression and localization were not clear. Since ER stress was not directly caused by loss of NGLY1 in many models, identification of the direct targets of NGLY1 and their contribution to the pathogenesis of NGLY1 deficiency is desired. For locations and types of the NGLY1 mutation for CDDG, almost all missense mutations were in the central transglutaminase domain, which might affect the de-glycosylating enzyme activity of NGLY1. However, a great portion of the missense mutations could result in the loss of mRNA or protein detected by quantification PCR or Western blot. The underlying mechanisms have still not been revealed. It has been reported that many cellular pathways could be disturbed by the loss of NGLY1, such as NFE2L1 pathway, Creb1/Atf1-AQP pathway, BMP pathway, AMPK pathway, SLC12A2 ion transporter, etc. These might be the underlying reasons for a constellation of clinical phenotypes of NGLY1 deficiency. Since many pathways function specifically and at different development stages, these make the treatment much more challenging. 

## Data Availability

All other data are available on request.

## Supplementary Materials

The following supporting information can be downloaded at: https://www.mdpi.com/article/10.3390/nu14091690/s1, Table S1: Properties of NGLY1.xlsx; Table S2: Reported patients with NGLY1 mutations.xlsx; Table S3: Functional prediction for missense mutations by missense3D.xlsx).
